# The Effect of FTY720 on Sphingolipid Imbalance and Cognitive Decline in Aged EFAD Mice

**DOI:** 10.3233/ADR-230053

**Published:** 2024-09-27

**Authors:** Qian Luo, Simone M. Crivelli, Shenghua Zong, Caterina Giovagnoni, Daan van Kruining, Marina Mané-Damas, Sandra den Hoedt, Dusan Berkes, Helga E. De Vries, Monique T. Mulder, Jochen Walter, Etienne Waelkens, Rita Derua, Johannes V. Swinnen, Jonas Dehairs, Mario Losen, Pilar Martinez-Martinez

**Affiliations:** a Department of Psychiatry and Neuropsychology, School for Mental Health and Neuroscience, Maastricht University, Maastricht, The Netherlands; b Institute of Neuroregeneration and Neurorehabilitation, Qingdao University, Qingdao, China; c Department of Physiology, University of Kentucky College of Medicine, Lexington, KY, USA; d Department of Internal Medicine, Laboratory Vascular Medicine, Erasmus MC University Medical Center, Rotterdam, The Netherlands; e Department of Organic Chemistry, Slovak University of Technology, Bratislava, Slovak Republic; f Department of Molecular Cell Biology and Immunology, Amsterdam Neuroscience, Amsterdam UMC Vrije Universiteit, Amsterdam, The Netherlands; g Department of Neurology, University Hospital Bonn, University of Bonn, Bonn, Germany; h Laboratory of Protein Phosphorylation and Proteomics, KU Leuven, Leuven, Belgium; i Laboratory of Lipid Metabolism and Cancer, KU Leuven, Leuven, Belgium

**Keywords:** Alzheimer’s disease, APOE4, ceramide, FTY720, sphingolipid

## Abstract

**Background:**

During Alzheimer’s disease (AD) progression, there is a decline in the bioactive sphingolipid sphingosine-1-phosphate (S1P). Previous research showed that FTY720, an S1P mimetic, prevented cognitive decline and reduced ceramide levels in transgenic mice with familial AD carrying the human APOE4 gene (E4FAD) at 6–7 months of age.

**Objective:**

The objective of this study is to explore the protective effects of FTY720 at late-stage AD.

**Methods:**

Male mice aged 9.5 to 10.5 months were orally administered FTY720 (0.1 mg/kg) via oral gavage for 6 weeks. A pre-test of water maze was used for evaluating the pathological status. After 4 weeks of administration, memory, locomotion, and anxiety were assessed. Cortex samples were analyzed for amyloid-β (Aβ) and sphingolipid levels.

**Results:**

Compared with APOE3 mice, APOE4, E3FAD and E4FAD mice exhibited significant memory deficits. After 6 weeks administration, FTY720 did not alleviate memory deficits in EFAD mice. Lipid analysis revealed that S1P was significantly reduced in EFAD mice (E3FAD or E4FAD) compared to controls (APOE3 and APOE4). Ceramide level alterations were predominantly dependent on APOE isoforms rather than AD transgenes. Interestingly, Cer (d18 : 1/22 : 1) was elevated in APOE4 mice compared to APOE3, and FTY720 reduced it.

**Conclusions:**

E4FAD and APOE4 mice exhibited significant spatial memory deficits and higher ceramide concentrations compared to APOE3 mice. FTY720 did not reverse memory deficits in E4FAD and APOE4 mice but reduced specific ceramide species. This study provides insights into the association between sphingolipids and APOE4 in advanced AD stages, exploring potential therapeutic targeting of sphingolipid metabolism.

## INTRODUCTION

Alzheimer’s disease (AD), the most common type of dementia, is marked by the accumulation of extracellular amyloid-β protein (Aβ), intracellular deposition of hyperphosphorylated tau protein in neurofibrillary tangles, neuroinflammation, and lipid alterations.^1 - 4^ However, the molecular mechanism driving neurodegeneration in AD remains uncertain.

Bioactive sphingolipids, including ceramide, sphingomyelin, and sphingosine 1-phosphate (S1P), serve as structural components of the cell membranes and play crucial roles in regulating various cellular processes in the central nervous system.^5^ Ceramide, the central metabolite of the sphingolipid pathway, has been implicated in apoptosis, while S1P has been reported to promote cell survival and differentiation.^6 - 8^ Ceccom et al. observed that downregulation of sphingosine-1-phosphate kinase 1 (SphK1) or upregulation of S1P lyase (SPL) reduced S1P synthesis in AD brains.^9^ Recent research has highlighted the potential of S1P in reducing Aβ accumulation, secretion, aggregation, and in attenuating the activity of β-site APP cleaving enzyme 1 (BACE1).^10 - 12^ Furthermore, deficiencies in SPL have been associated with accumulation of full-length APP and amyloidogenic C-terminal fragments,^13^ suggesting that modulating S1P levels could hold promise for AD therapy.

Fingolimod (FTY720), a sphingosine analog approved for the treatment of multiple sclerosis, is metabolized *in vivo* to generate FTY720-P. Recent studies have revealed the protective functions of FTY720 in AD models. FTY720 reduces Aβ production by inhibiting *γ*-secretase and attenuates Aβ-induced neurotoxicity by suppressing caspase-dependent pathways.^14 - 16^ Additionally, FTY720 mitigates memory deficits in young APP/PS1 transgenic mice and reduces microglia activation in the 5xFAD transgenic mouse model.^12,17,18^ The ratio of S1P/sphingosine is 2.5-fold higher in the hippocampus of APOE2 carriers compared to APOE4 carriers, which represents the strongest known genetic risk factor for late-onset AD.^19^ Studies have also reported that FTY720 influences the gene transcription of sphingolipid enzymes, including SphKs.^20,21^ Moreover, our study demonstrated that FTY720 administration prevents Aβ accumulation and spatial memory decline in 6 to 7-month-old E4FAD mice, while reducing the levels of long-chain ceramides in APOE4-carrying mice.^22^ These results encouraged us to perform a follow-up study to investigate the protective effects of FTY720 at an advanced stage of AD pathology.

In this study, APOE4-carrying mice exhibited significant memory impairments, which were not inhibited by FTY720. Additionally, a greater variety of ceramides increased in APOE4 mice compared to APOE3 mice. Among all the changes observed in sphingolipids, the level of Cer (d18 : 1/22 : 1) was elevated in APOE4-carrying mice, and FTY720 lowered it. Taken together, these results underscore the importance of APOE isoforms as major factors in selecting AD treatment strategies involving sphingolipid signaling.

## MATERIALS AND METHODS

### Drug preparation and animals

The drug FTY720 was kindly provided by Barbara Nuesslein-Hildesheim, Novartis Pharma AG Basel, Switzerland in powder form. The drug was dissolved in water and stored at 4°C as a stock (1 mg/ mL).

All experiments were performed following the laws, rules, and guidelines of the Animal Welfare Committee of Maastricht University. Animals were purchased from Dr. Mary Jo LaDu’s laboratory.^23^ The information of groups is described in Table 1. FTY720 (0.1 mg/kg/day) was given by oral gavage for 6 consecutive weeks for 9.5–10.5-month-old male mice.

**Table 1:** Number of animals in the experimental groups

	Control mice		EFAD mice	
	APOE3	APOE4	E3FAD	E4FAD
Vehicle	19	16	23	16
FTY720	18	13	19	17

### Behavioral tasks

Memory, locomotion, and anxiety levels were assessed for 4 weeks post treatment.

### Water maze (WM)

WM was arranged to assess spatial memory as previously explained.^24,25^ The first WM test was conducted as a pre-test before the administration of FTY720. After a four-week treatment period, the second WM test was performed as a post-test. Briefly, all animals underwent four consecutive days of training to locate and remember the hidden platform. On the fifth day, the mice were subjected to a final 60-s probe trial. During this trial, the latency to reach the platform and the time spent in the target quadrant were recorded using the EthoVision XT tracking system (Noldus Information Technology).

### Y-maze spontaneous alternation (AYM)

The AYM test consists of three arms of equal length arranged at the same angles. Mice were placed randomly in one of the three arms of the Y-maze and were left free to explore the arena for 6 min. The number of entries and alternations were recorded over a 3-min period. The percentage of consecutive alternations was calculated using the formula: the percentage of consecutive alternation = number of successful consecutive visits to 3 different arms / (number of arm entries –2) *100%.

### Open field (OF)

The OF task was assigned to assess locomotion as previously described.^26^ The mice were placed in the center of the arena, and the total distance traveled was recorded using the EthoVision XT tracking system (Noldus Information Technology).

### Elevated zero-maze (EZM)

The EZM test was conducted to assess anxiety levels in the mice. The mice were initially placed in an open arm of the circular runway maze and allowed to explore freely. The time spent in the closed arm, open arm, and the distance traveled were recorded using an infrared video camera connected to the EthoVision XT video tracking system (Noldus Information Technology). Percentage of time spent in the open arms was corrected for latency to first closed arm entry.^25^

### Lipidomic analysis

Prior to lipidomic measurement, the cortex was powdered, aliquoted and weighed.^22^ Both sample preparation and measurement were described previously.^27,28^ Briefly, 800μL water was added to the cortex powder of each mouse and then homogenized with a Precellys 24 Tissue Homogenizer, of which 700μL was used for lipid extraction. SPLASH LIPIDOMIX Mass Spec Standard (#330707, Avanti Polar Lipids) was added into the extract mix. The mass spectrometry measurement was performed by liquid chromatography electrospray ionization tandem mass spectrometry (LC-ESI/MS/MS). The sphingolipid fatty acyl chains were detected in lipidomic analysis: 14 : 0, 14 : 1, 16 : 0, 16 : 1, 16 : 2, 18 : 0, 18 : 1, 18 : 2, 18 : 3, 20 : 0, 20 : 1, 20 : 2, 20 : 3, 20 : 4, 20 : 5, 22 : 0, 22 : 1, 22 : 2, 22 : 4, 22 : 5, and 22 : 6. Lipid species signals were calculated with Python Molmass 2019.1.1 and were quantified based on standard internal signals. The abundance of lipid species is reported as a percentage of their respective classes, using shorthand notation for the lipids as per previously established guidelines.^29^

### Amyloid-β quantification assay

Aβ was extracted from the cortex through sequential centrifugation into three different fractions: aqueously soluble (Tris-buffered saline [TBS]), detergent-soluble (TBS buffer containing 1% Triton-X 100 [TBST]), and insoluble (70% formic acid [FA]) for a more comprehensive characterization of Aβ species present in biological samples. These fractions were then measured using a sandwich enzyme-linked immunosorbent assay (ELISA).^25^ In brief, a 96-well plate was coated with 1μg/mL of the 3D6 antibody^25^ overnight and subsequently blocked with 4% non-fat milk for 1 h. Next, the samples were added to the plate and incubated for 1 h. After incubation, the samples were treated with a biotinylated 20C2 antibody^25^ for another hour to bind the antigens. Following this, streptavidin-HRP was added for 1 h. Samples were then incubated with tetramethylbenzidine (TMB) substrate solution; to terminate the reaction, 50μL of stop solution was added to the plate. Finally, the plate was read at 450 nm using the PerkinElmer 2030 manager system.

### Statistics

All data were analyzed using IBM SPSS Statistics for Windows, version 27.0, and graphs were created with GraphPad Prism, version 9.0.0. An unpaired *t*-test was used to compare the group means of S1P levels in the brains of EFAD (E3FAD and E4FAD) and control (APOE3 and APOE4) mice. Additionally, an unpaired *t*-test was used to compare the means of Aβ levels in control or FTY720-treated EFAD mice (E3FAD and E4FAD). Lipidomic data were analyzed using volcano plots generated with GraphPad Prism to highlight differences based on AD or APOE isoforms.

One-way ANOVA was used to analyze the effect of genotype (APOE3, APOE4, E3FAD, and E4FAD) on spatial memory assessed in the WM pre-test. To test the effect of genotype or the drug FTY720 on spatial memory, the success rate in finding the hidden platform was determined using the Kaplan-Meier curve with the Log-rank (Mantel-Cox) test, as recommended.^30^ Two-way ANOVA was performed to study the main effects and the interactions between genotype (APOE3, APOE4, E3FAD, and E4FAD) and treatment (Control and FTY720) followed by Fisher’s least significant difference or Tukey’s multiple comparisons test. *p* < 0.05 was regarded as a significant change.

## RESULTS

### APOE4 impairs memory function in 9.5–10.5-month-old male mice

**Fig. 1:**
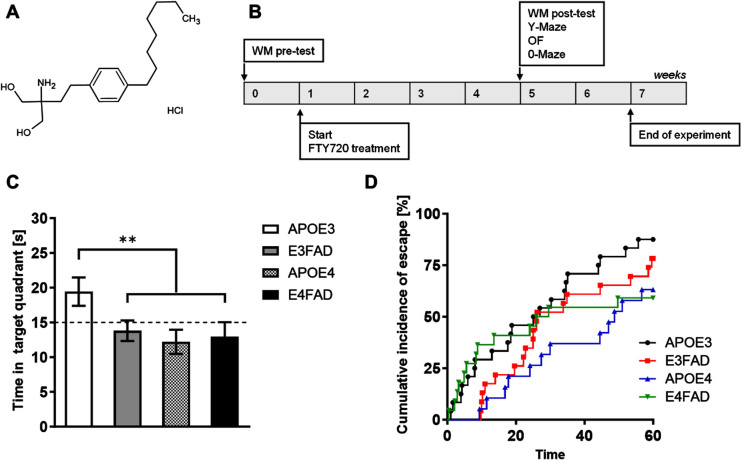
Experimental timeline and memory performance results before treatment initiation. A) The chemical structure of FTY720. B) Timeline of the experiment, illustrating behavioral evaluations (pre- and post-test) and the start of FTY720 treatment. During the pre-test stage, only the Water Maze (WM pre-test) was conducted on animals aged 9.5 to 10.5 months. After 4 weeks of treatment, animals underwent the WM post-test, as well as the AYM, EYM, and OF tests. C) Bar graph showing the time spent in the target quadrant, expressed in seconds (one-way ANOVA, Tukey’s multiple comparisons test, **p < 0.01). Each bar represents the mean±SEM of 19–25 animals/group. D) Kaplan-Meier estimate curves for each genotype, depicting the percentage of successful attempts to find the hidden platform during the test trial (*p* < 0.05, Log-rank test).

The chemical structure of FTY720 and experimental schematic illustration of this study are illustrated in Fig. 1A and 1B. To assess spatial memory deficits before the beginning of the treatment, we performed a WM test at baseline (WM pre-test). Compared to APOE3 mice, APOE4 and E4FAD mice spent significantly less time in the target quadrant during the probe trial of the WM (*p* = 0.0286). Latency outcomes were analyzed using Cox regression modeling to account for potential biases arising from animals failing to escape within the given time frame (Fig. 1C).^30^ The results indicated that the majority of APOE3 mice successfully located the platform, whereas only approximately 50% of APOE4 and E4FAD mice completed the task correctly (Fig. 1D) (HR = 2.206, 95% CI 1.102 to 4.418, *p* = 0.0255).

Our data suggested that the APOE4 gene has a detrimental effect on memory with aging and in association with AD transgenes.

### Sphingolipid levels in APOE3, E3FAD, APOE4, and E4FAD mice

**Fig. 2:**
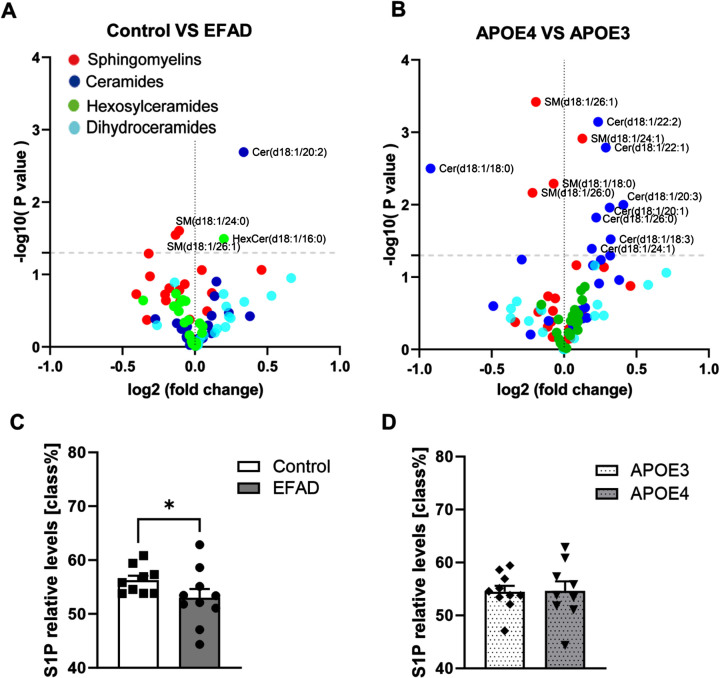
LC-ESI/MS/MS measurements of ceramide, sphingomyelin, dihydroceramide, and monohexosyl-ceramide levels in the cortex. A) Volcano plot depicting sphingolipid changes between AD (E3FAD, *N* = 5; E4FAD, *N* = 5) and control (APOE3, *N* = 5; APOE4, *N* = 4) groups. B) Volcano plot showing differences in sphingolipid levels between APOE4 mice (APOE4, *N* = 4; E4FAD, *N* = 5) and APOE3 mice (APOE3, *N* = 5; E3FAD, *N* = 5). C, D) Changes in S1P levels between control and EFAD mice, and between APOE3 and APOE4 mice, respectively. Data are expressed as mean±SEM. *p < 0.05.

To explore the sphingolipid changes in APOE3, E3FAD, APOE4, and E4FAD mice, ceramides (Cer), dihydroceramide, sphingomyelins (SM), and monohexyl-ceramides (HexCer) were measured by lipid chromatography-electrospray ionization tandem mass spectrometry (LC-ESI/MS/MS). First, to understand the effect of AD status, the data was separated into control (APOE3 and APOE4) and EFAD groups (E3FAD and E4FAD). We found that Cer (d18 : 1/20 : 2) and HexCer (d18 : 1/16 : 0) were increased in EFAD mice, while SM (d18 : 1/26 : 1) and SM (d18 : 1/24 : 0) were reduced (Fig. 2A). Next, to explore the effects of APOE genotypes, the data was separated into APOE3 (APOE3 and E3FAD) and APOE4 (APOE4 and E4FAD) groups. The results demonstrated that almost all ceramide species were increased in APOE4 mice, while SM (d18 : 1/18 : 0), SM (d18 : 1/26 : 0), and SM (d18 : 1/26 : 1) were decreased (Fig. 2B). Additionally, S1P levels were significantly reduced in the EFAD mice compared to the control mice (Fig. 2C), but they were unaffected by the APOE background (Fig. 2D).

These results indicated that the APOE isoforms had a greater impact on ceramide levels than the AD transgene, while reduction of S1P levels was associated with AD transgene more than the APOE isoforms.

### Analysis of behaviors in FTY720 treated E3FAD and E4FAD mice

**Fig. 3:**
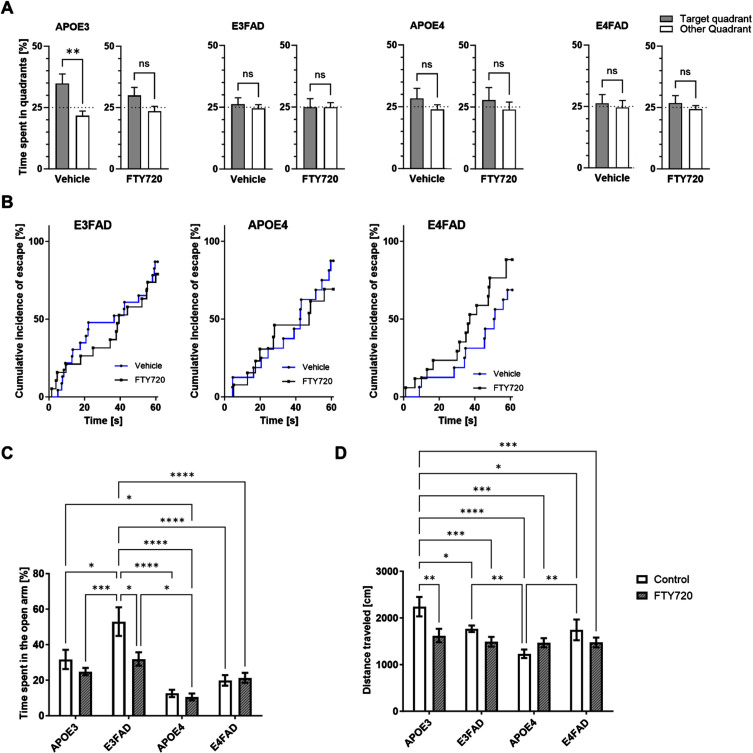
Treatment effects of FTY720 on behavioral tests. A) Bar graph showing the time spent in the target quadrant and other quadrants, expressed as a percentage of time. An unpaired *t*-test was used to compare the mean time spent in the target quadrant versus the mean time spent in other quadrants (**p < 0.01). Each bar represents the mean±SEM of 13–23 animals per group. B) Kaplan-Meier estimate curves for each genotype, depicting the percentage of successful attempts to find the hidden platform during the test trial (*N* = 13–23 animals per group). C) Bar graph showing the percentage of time spent in the open arms of the EZM (*N* = 13–17 animals per group; ANOVA, Tukey post-hoc *p < 0.05, **p < 0.01, ***<0.001, *p*< ****). E) Bar graph showing the distance traveled was measured during the OF test. Each bar represents mean±SEM of 13–17 animals per group (ANOVA, Tukey post-hoc *p < 0.05, **p < 0.01, ***<0.001, *p*< ****).

To determine whether FTY720 can reverse memory, anxiety, and locomotion deficits, the animals underwent WM, AYM, EZM, and OF tests. Our results showed that in the WM post-test, only the APOE3 animals treated with vehicle spent significantly more time in the target quadrant, while all other groups spent less or an equal amount of time compared to the 25% chance level (Fig. 3A). When analyzing the escape latency in the WM, we found that FTY720 treatment slightly improved the performance of E4FAD mice by approximately 1.8-fold, though this was not statistically significant (HR = 1.811, 95% CI 0.8248 to 3.975, *P* = 0.1389) (Fig. 3B). Additionally, FTY720 treatment did not significantly affect spatial memory as assessed by the AYM (Supplementary Figure 1A).

Consistent with our previous findings,^22^ mice carrying the APOE4 allele exhibited increased anxiety (Fig. 3C). E3FAD mice showed reduced anxiety compared to APOE3 mice, and FTY720 normalized this behavior in E3FAD (Fig. 3C). The OF test revealed that APOE3 mice traveled longer distances compared to other genotypes (Fig. 3D). E4FAD mice showed increased locomotion activity compared to APOE4 mice (Fig. 3D). FTY720 treatment significantly reduced locomotion activity in APOE3mice.

Our data indicate that APOE4 increases anxiety behavior and reduces mobility. FTY720 could not reverse the spatial memory deficits found in the E3FAD, APOE4, and E4FAD groups. The drug normalized the reduced anxiety observed in the E3FAD group.

### FTY720 changed the sphingolipid levels in the cortex

**Fig. 4:**
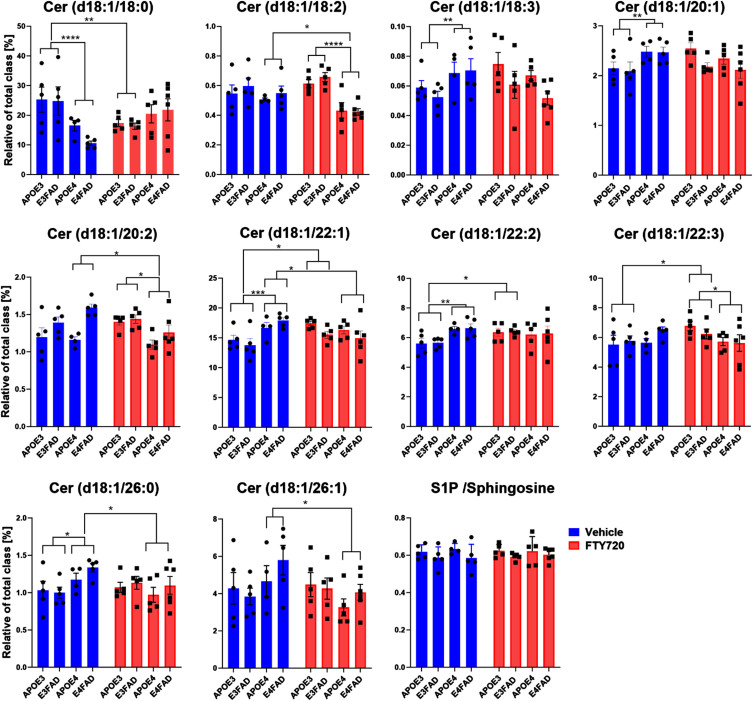
The effect of FTY720 on ceramides classified by the number of carbons of the acyl chain and S1P in the cortex. Bars represent the mean±S.E.M of Cer d18 : 1/18 : 0, Cer d18 : 1/18 : 2, Cer d18 : 1/18 : 3, Cer d18 : 1/20 : 1, Cer d18 : 1/20 : 2, Cer d18 : 1/22 : 1, Cer d18 : 1/22 : 2, Cer d18 : 1/22 : 3,Cer d18 : 1/26 : 0, Cer d18 : 1/26 : 1, and S1P / Sphingosine ratio measured in the cortex. Each bar represents mean±SEM of 5 animals. (ANOVA, LSD post-hoc, *p < 0.05, **p < 0.01). (^*^p < 0.05, ^**^p < 0.01, ^***^p < 0.001, ^****^p < 0.0001).

To analyze the effect of FTY720 on sphingolipid levels, LC-ESI/MS/MS was employed. We observed that FTY720 predominantly decreased ceramide levels in treated APOE4 mice compared to the untreated group, specifically Cer (d18 : 1/18 : 2), Cer (d18 : 1/20 : 2), Cer (d18 : 1/26 : 0), and Cer (d18 : 1/26 : 1), as illustrated in Fig. 4A. Conversely, FTY720 increased the levels of Cer (d18 : 1/18 : 0), Cer (d18 : 1/22 : 2), and Cer (d18 : 1/22 : 3) in treated APOE3 mice. Notably, Cer (d18 : 1/22 : 1) was elevated in APOE4 mice compared to APOE3, and FTY720 treatment mitigated this elevation. Additionally, APOE4 mice exhibited heightened levels of sphingomyelin, dihydroceramide, and monohexosyl-ceramide in the cortex compared to APOE3, while no significant differences were observed between FTY720-treated and untreated groups (Fig. 4B). The level of S1P did not exhibit significant differences. In summary, FTY720 had an impact on ceramide levels in EFAD mice depending on APOEisoforms.

### Analysis of amyloid levels in FTY720 treated E3FAD and E4FAD mice

**Fig. 5:**
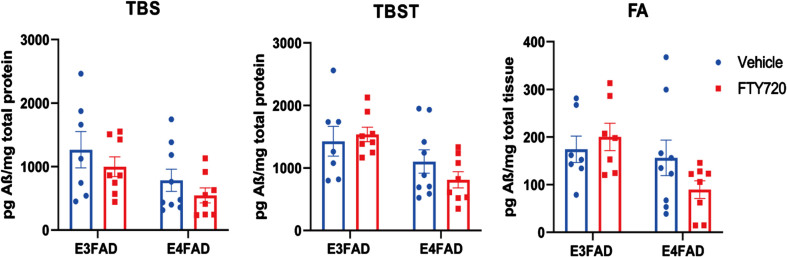
Quantification of Aβ extracted from brains of E3FAD and E4FAD animals. Aβ was extracted from brains of E3FAD and E4FAD animals in three fractions: TBS, TBST, and formic acid (FA), and quantified by ELISA. The bar graphs represent the mean±SEM. An unpaired *t*-test was used to compare E3FAD or E4FAD animals treated with Vehicle or FTY720. Each bar represents the mean±SEM of 7–10 animals.

In AD brains, higher levels of Aβ are correlated with the severity of cognitive impairment.^31^ To investigate the effect of FTY720 on Aβ accumulation in the brain, Aβ was collected in three different protein fractions, including TBS, TBST, and FA. In the TBS-T and FA fraction, we observed a 30 and 40% reduction, respectively, in the E4FAD groups treated with FTY720 when compared to vehicle (Fig. 5). However, the t-test was not statistically significant.

This data suggests that FTY720 might mildly reduce Aβ levels in the late stage of AD, especially in APOE4 carriers.

## DISCUSSION

In our previous study, we reported that FTY720 prevented cognitive decline, decreased Aβ levels and reduced accumulation of toxic long chain ceramides in 6–7 month old E4FAD mice.^22^ These findings prompted us to investigate whether FTY720 could mitigate AD pathophysiology in the late stage. Here, we found that mice carrying APOE4 exhibited worse spatial memory compared to APOE3-carrying mice at 9.5 to 10.5 months of age. Additionally, we observed that APOE isoforms had a more significant impact on sphingolipid metabolism than the AD transgenes, particularly leading to elevated levels of long-chain ceramides. Nevertheless, the levels of S1P were significantly reduced in the EFAD mice (E3FAD or E4FAD) compared to the control mice (APOE3 and APOE4) and the levels of Cer (d18 : 1/22 : 1) were selectively elevated in APOE4 mice. Although FTY720 treatment slightly improved the spatial memory performance of E4FAD mice, the effect was not statistically significant. Interestingly, FTY720 reduced the most insoluble forms of Aβ (TBS-T and FA) levels by 30–40%, although this reduction was not statistically significant. Additionally, following FTY720 administration, ceramide levels, such as Cer (d18 : 1/18 : 2), Cer (d18 : 1/20 : 2), Cer (d18 : 1/22 : 1), Cer (d18 : 1/26 : 0), and Cer (d18 : 1/26 : 1), were significantly reduced.

APOE4 is considered one of the greatest genetic risks for late-onset AD, yet the molecular mechanisms by which APOE4 contributes to the disease remain unclear.^32^ Human studies have shown that both heterozygous and homozygous APOE4 exhibit poorer cognition and a higher risk of dementia with aging.^33^ Now it has been proposed that APOE4 homozygosity might be considered as a distinct genetic form of AD.^34^ Couttas et al. reported that S1P production in the brain is linked to APOE4 genes and is compromised early in the AD process.^35^ They observed a decrease in the ratio of S1P to sphingosine, particularly in the CA1 region of the hippocampus and the middle temporal gyrus, especially among individuals carrying the APOE4 allele. Similarly, in our animal models, we found that the APOE4 mice had impaired spatial memory compared to APOE3 carriers and that the S1P levels were diminished in EFAD mice. However, this was independent from APOE background.

Our previous study indicated that sex, age, and APOE4 status were important factors modulating sphingolipid metabolism in AD. Among these factors, sex exerted a stronger influence on ceramide changes, with only Cer (d18 : 1/24 : 0) significantly affected by APOE4 or AD state.^36^ In this study, we measured sphingolipid changes in 11–12-month-old male mice, revealing increases in Cer (d18 : 1/18 : 3), Cer (d18 : 1/20 : 0), Cer (d18 : 1/22 : 0), Cer (d18 : 1/22 : 1), Cer (d18 : 1/22 : 2), Cer (d18 : 1/24 : 1), and Cer (d18 : 1/26 : 0) in APOE4 mice. Considering these findings alongside our previous publications, it appears that the differences in ceramide species between APOE3 and APOE4 increase with the aging of the animals.

Previous studies have demonstrated the beneficial effects of FTY720 in AD, including improvements in spatial memory, reduction of Aβ levels, and modulation of neuroinflammatory markers in rat and 3xTg-AD models.^17,37^ Similarly, our recent findings showed that FTY720 prevented memory impairments in 6–7-month-old E4FAD mice.^22^ However, in this study, FTY720 increased did not rescue memory deficits in EFAD mice. It is possible that the 6-week treatment duration in this study was insufficient to elicit significant behavioral changes compared to longer treatments or interventions at younger ages.^22,37^ Interestingly, FTY720 normalized reduced anxiety in E3FAD mice. In our previous study, we reported that FTY720 affected anxiety behavior in female mice carrying APOE4 alleles. Like these findings, APOE4 mice in this study showed a tendency to spend significantly less time in the open arms of the EZM test. However, unlike our previous findings, APOE4 mice in this study did not respond to the treatment, while E3FAD mice showed a positive response. The difference in response may be attributed to variations in age or sex between this study and our earlier findings.

Increasing evidence highlights the impact of FTY720 on sphingolipid metabolism. Firstly, FTY720 has been shown to upregulate hippocampal ceramide synthase in young mice and sphingomyelin synthases SGMS1/2 in older mice.^20^ Secondly, FTY720 increases the activity of SphKs, promoting the conversion of sphingosine into S1P and upregulating anti-apoptotic Bcl expression.^21^ Thirdly, our previous study demonstrates that administration of FTY720 decreases ceramide levels in 6–7-month-old E4FAD mice.^22^ Specifically, we observed reductions in Cer (d18 : 1/18 : 2), Cer (d18 : 1/26 : 0), and Cer (d18 : 1/26 : 1) in APOE4 carriers following FTY720 treatment. Importantly, we noted an elevation of Cer (d18 : 1/22 : 1) in APOE4 mice, which was attenuated by FTY720 administration. Further experiments are warranted to elucidate the mechanisms underlying FTY720-induced changes in Cer (d18 : 1/22 : 1) levels in APOE4 mice.

In conclusion, our study underscores the significance of APOE4 isoforms in both memory decline and sphingolipid metabolism in mice. Specifically, we observed that APOE4 tended to induce greater changes in ceramides compared to sphingomyelin or other species. Moreover, our findings indicate that FTY720 had a more pronounced effect in reducing ceramides rather than sphingomyelins. These results suggest a potential connection between APOE4 and FTY720 in modulating ceramides in the context of AD. However, further studies are warranted to elucidate and clarify this connection more comprehensively.

## AUTHOR CONTRIBUTIONS

Qian Luo (Data curation; Formal analysis; Methodology; Writing – original draft); Simone M. Crivelli (Conceptualization; Data curation; Formal analysis; Supervision; Writing – original draft); Shenghua Zong (Methodology; Supervision; Writing – review & editing); Caterina Giovagnoni (Methodology; Writing – review & editing); Daan van Kruining (Data curation; Formal analysis; Methodology; Writing – review & editing); Marina Mané-Damas (Methodology; Writing – review & editing); Sandra den Hoedt (Data curation; Formal analysis; Methodology; Writing – review & editing); Dusan Berkes (Conceptualization; Funding acquisition; Methodology; Writing – review & editing); Helga E. De Vries (Conceptualization; Funding acquisition; Writing – review & editing); Monique T. Mulder (Conceptualization; Funding acquisition; Writing – review & editing); Jochen Walter (Conceptualization; Funding acquisition; Writing – review & editing); Etienne Waelkens (Data curation; Methodology; Writing – review & editing); Rita Derua (Data curation; Methodology; Writing – review & editing); Johannes V. Swinnen (Data curation; Methodology; Writing – review & editing); Jonas Dehairs (Data curation; Methodology; Writing – review & editing); Mario Losen (Conceptualization; Funding acquisition; Project administration; Writing – original draft; Writing – review & editing); Pilar Martinez-Martinez (Conceptualization; Funding acquisition; Methodology; Project administration; Writing – original draft; Writing – review & editing).

## Supplementary Material

Supplementary Material

## Data Availability

The data supporting the findings of this study are available on request from the corresponding author.
