# Supplementary material for: The Effect of FTY720 on Sphingolipid Imbalance and Cognitive Decline in Aged EFAD Mice
Source: J Alzheimers Dis Rep. 2024 Sep 27;8(1):1317–27. doi: 10.3233/ADR-230053 (PMC11491960; doi:10.3233/ADR-230053)
Supplement: Supplementary Material [file adr-8-adr230053-s001.pdf]

Supplementary Material

The Effect of FTY720 on Sphingolipid Imbalance and Cognitive Decline in Aged EFAD Mice

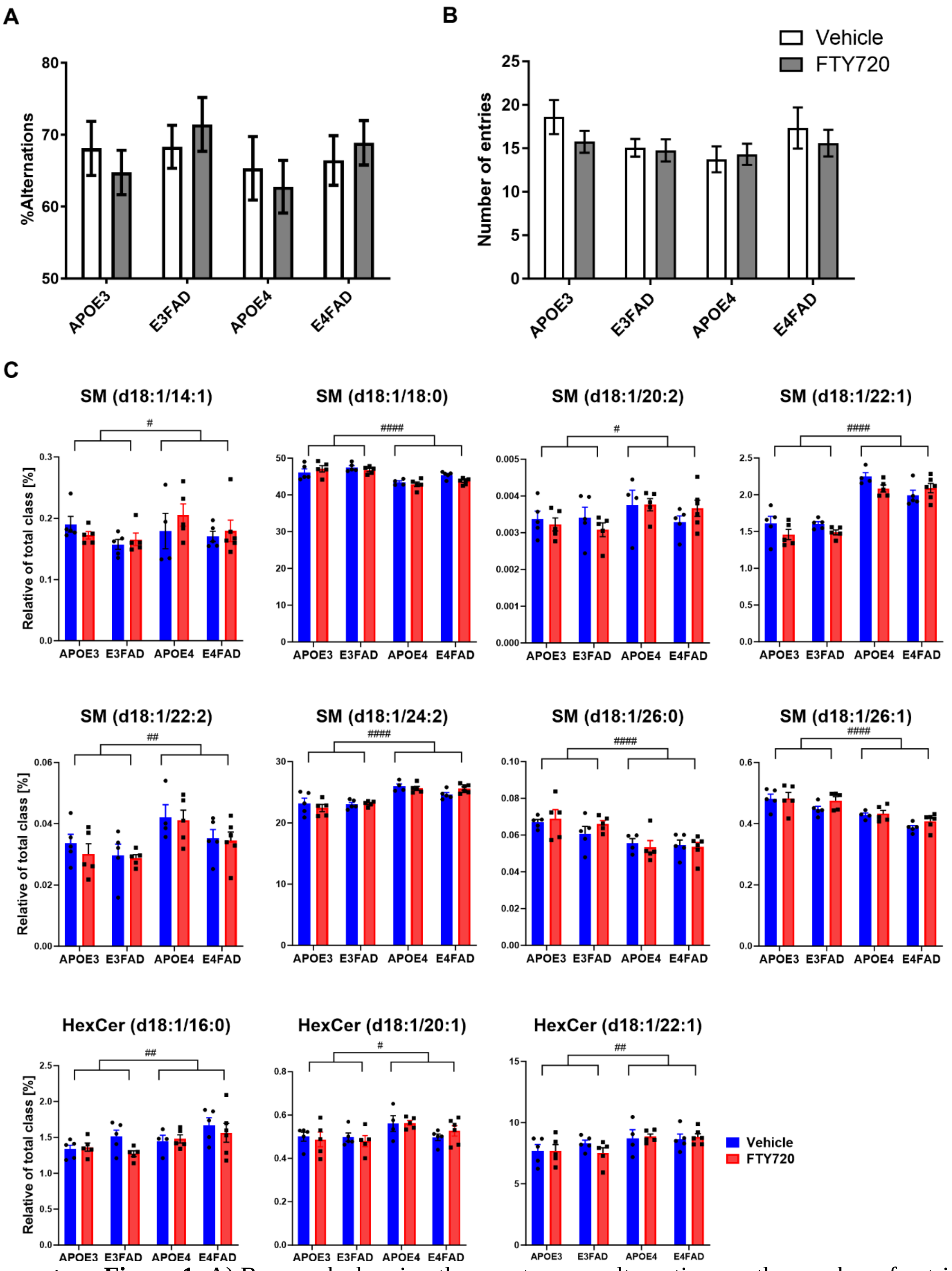

Supplementary Figure 1. A) Bar graph showing the spontaneous alternations or the number of entries (B) in the first 8 triads, calculated in AYM (N=13-17 animals / group). C) Sphingomyelins (SM) and monohexosyl-ceramides (HexCer) classified by the number of carbons of the acyl chain measured by LC-ESI/MS/MS in the cortex were. (ANOVA main effect of APOE4 $^{\#}p<0.05$, $^{\#\#}p<0.01$, $^{\#\#\#}p<0.001$, $^{\#\#\#\#}p<0.0001$).
